# Food literacy assessment tools for adults: a scoping review of dimensional evolution, cultural adaptation, and potential implications for nursing-related practice

**DOI:** 10.3389/fnut.2026.1871292

**Published:** 2026-10-07

**Authors:** Yuxiao Ren, Chenqi Su, Hongbo Qiu, Runhao Jin

**Affiliations:** School of Nursing, Yanbian University, Yanji, Jilin, China

**Keywords:** adults, assessment tools, cultural adaptation, food literacy, scoping review

## Abstract

**Introduction:**

An unhealthy diet is a major modifiable risk factor for non-communicable diseases. Higher levels of food literacy facilitate the adoption of healthy dietary behaviours; therefore, the development and validation of assessment tools are crucial for public health and clinical practice. However, existing assessment tools are fragmented, and there is a lack of systematic analysis regarding dimensional evolution, cross-cultural adaptation, and practical implementation pathways.

**Materials and methods:**

This scoping review employed the Arksey and O’Malley framework along with PRISMA-ScR guidelines. Seven databases (PubMed, Web of Science, Embase, Wanfang, SinoMed, CNKI, and VIP) were searched for studies published between January 1, 2015, and November 16, 2025. Using descriptive content analysis, we synthesized evidence regarding how these tools have evolved, how they are adapted across cultures, and how they may be applied in practice.

**Result:**

A total of 28 studies involving 21 assessment tools were included; 13 were newly developed tools, and eight were culturally adapted or validated tools. The analysis identified three major findings: (1) Food literacy assessment tools commonly incorporated four core domains: skills, knowledge, behaviours, and attitudes, with some instruments extending beyond individual competencies toward broader social, environmental, and food-system-related dimensions; (2) Cultural adaptation strategies were descriptively summarized into three categories: language and semantic adaptation, structural and measurement adaptation, and conceptual and value embedding, while deeper integration of culturally embedded meanings remained limited; (3) Most tools had limited evidence regarding implementation in real-world community or clinical settings, particularly concerning the linkage between assessment results and subsequent interventions or nursing practice.

**Conclusion:**

Food literacy assessment tools have continued to expand in dimensional scope and cultural adaptation approaches. However, many tools remain focused on measuring static individual competencies and provide limited insight into how food-related capabilities are applied within real-life contexts. Future research should prioritize contextualized assessment approaches, implementation studies in community and clinical settings, and evaluation of the relationships between assessment results and health outcomes. Strengthening collaboration among nurses and other healthcare professionals may facilitate the translation of food literacy assessment into individualized and context-sensitive interventions.

**Systematic review registration:**

OSF, registration DOI: 10.17605/OSF.IO/3T6M5

## 1 Introduction

An unhealthy diet is a significant modifiable risk factor for non-communicable diseases globally (1). Despite decades of global public health initiatives aimed at disseminating nutritional knowledge, such as the risks associated with sugar intake and chronic diseases, and notably increasing public awareness, improved knowledge has not fully translated into behavioural change (2–5). How to convert nutritional knowledge into sustainable healthy dietary behaviours remains a central unresolved challenge within public health and clinical practice (6). Although various assessment tools exist to guide this transformation, there is no scoping review summarizing their dimensional evolution, cultural adaptation or effective integration into nursing practice.

Traditionally, food literacy has been framed in terms of knowledge, skills, and behaviours related to the planning, purchasing, preparation, and consumption of healthy meals and snacks, which, in itself, consume a significant part of dietary behaviour (7, 8). Cullen et al. further developed Vidgen’s definition by incorporating environmental, social, economic, and cultural considerations as far as the health of the personal diet is concerned. The integrated definition of food literacy also enhances food literacy through the addition of nonfunctional aspects, including the assessment and critique of food knowledge and the comprehension of food choices’ effects on the societal and environmental systems. This is the development of food literacy to critical and functional levels (9, 10). Thus, food literacy can be generalized as a multi-faceted notion that involves the knowledge and skills, behaviours, and attitudes required to eat healthily. The evaluation process needs accurate identification strategies that permit the identification of food literacy skills in the clinical context since the skills have a direct influence on dietary decisions and health outcomes (11). This is possible through the development of assessment tools that provide the appropriate results and full functionality.

Food literacy assessment should be conceptually distinguished from nutritional assessment and nutritional risk screening. Nutritional assessment primarily evaluates an individual’s nutritional status, dietary intake, and nutrition-related needs, whereas nutritional risk screening focuses on identifying individuals at risk of malnutrition or adverse nutritional outcomes (12). In contrast, food literacy assessment evaluates the knowledge, skills, behaviours, and attitudes that influence how individuals acquire, interpret, evaluate, and apply food-related information in daily life (13). Therefore, food literacy assessment does not aim to diagnose nutritional problems or determine nutritional treatment needs; rather, it may provide complementary information regarding behavioural and capability-related factors underlying dietary practices.

Although food literacy assessment is not equivalent to nutritional assessment or medical nutrition therapy, it may provide additional information that is relevant to patient education and behavioural support. Individuals may understand dietary recommendations but still experience difficulties translating this knowledge into daily food practices due to limited cooking skills, difficulties interpreting food information, insufficient confidence, or contextual barriers (14). By identifying these capability-related barriers, food literacy assessment may complement traditional nutritional evaluation and help healthcare professionals better understand factors affecting dietary behaviour.

Food literacy assessment should be distinguished from comprehensive nutritional assessment and medical nutrition therapy, which require specialized professional expertise. Registered dietitians and clinical nutrition professionals play a central role in nutrition assessment, individualized nutrition intervention, and therapeutic dietary counselling. Nurses frequently engage in patient education, chronic disease management, discharge preparation, and follow-up care, which may provide opportunities to identify barriers affecting patients’ ability to implement dietary recommendations (15). However, the involvement of nurses in nutrition-related care varies across healthcare systems and professional regulations. Therefore, the potential relevance of food literacy assessment to nursing should be understood as a complementary component of interdisciplinary care rather than a universal nursing responsibility. Food literacy assessment may therefore support nurses in identifying educational needs, reinforcing general dietary guidance, facilitating appropriate referral to nutrition professionals, and monitoring patients’ self-management, rather than replacing specialized nutritional assessment or dietary therapy (16, 17).

Research has affirmed that there is a high degree of connection between food literacy and healthy eating habits. An individual with higher food literacy is associated with better food-related knowledge and skills that result in healthier eating habits (18). In turn, the promotion of food literacy has been considered internationally as the key to supporting and maintaining healthy eating habits (19, 20). In this regard, the creation and validation of multi-dimensional and accurate food literacy assessment tools is essential to further research, policy, and correct clinical and population health interventions (21). Multi-dimensional means that food literacy contains multiple aspects that cannot be well represented by any single dimension, whereas precise means robust reliability and validity, which allows identifying the gaps in the food literacy of individuals or groups to conduct scientific follow-up and clinical interventions (22). Though Western countries have achieved much in defining food literacy and investigating the relationships between food literacy and nutrition and health, global progress remains unequal. Areas like the Middle East and North Africa are still at a tender age, underscoring the necessity for culturally localized research (23, 24).

Furthermore, despite its conceptual strength, the food literacy framework also has several challenges in the assessment field, including the level of quality of the tools used and measurement bias. By using these scientifically validated tools of assessment, it would be possible to monitor food literacy at the population level, plan and appraise the effectiveness of interventions and guide the policy and practice of the general population of various countries toward advances in health (18, 25). The food literacy assessment tools have now been developed in the international arena and are used by university students, low-income adults, diabetic adults, and the elderly population of the community. Nonetheless, there are a number of gaps in research: First, despite the many tools available, their evolutionary patterns have not been thoroughly examined; Secondly, no scoping review has examined the adaptation strategies and cross-cultural food literacy assessment tools., despite their engagement of profound cultural aspects of dietary patterns, sociocultural practices, as well as health belief systems (26, 27); Most importantly, the applicability of these tools in community and clinical practice remains unclear, limiting their translation from research to practice. Therefore, it is necessary to conduct an urgent scoping review to bring together existing evidence and to explain the knowledge field in this area.

Few reviews have synthesized existing food literacy assessment tools. Amouzandeh et al. conducted a scoping review of 12 adult food literacy assessment tools and compared their conceptual coverage based on the framework proposed by Vidgen and Gallegos, providing important insights into the validity, reliability, and conceptual alignment of existing measures (25). However, their review primarily focused on the measurement characteristics and conceptual foundations of available instruments. Since the publication of that review, additional food literacy assessment tools have been developed, validated, and adapted across diverse populations and cultural contexts. Consequently, several important questions remain unresolved, including how the dimensions of food literacy assessment tools have evolved, how cross-cultural adaptation strategies have been implemented, and how assessment findings may be translated into community and clinical practice. Therefore, a comprehensive synthesis integrating instrument development pathways, cultural adaptation processes, and potential practice applications is warranted. Unlike the earlier review, the present scoping review focuses on the evolution of assessment tools published since 2015, reported cross-cultural adaptation procedures, and potential pathways for translation into community, clinical, and interdisciplinary practice.

Nevertheless, evidence regarding the direct administration of food literacy assessment tools by nurses or the use of assessment results to guide nursing interventions remains limited. Therefore, the present review considers nursing as a potential translation context rather than an established implementation setting.

The consideration of nursing-related contexts in this review does not imply that food literacy assessment belongs exclusively to nursing practice. Rather, nursing represents one potential pathway through which assessment information may be translated into patient-centred care. Nurses frequently participate in health education, chronic disease management, care transitions, and follow-up support, where identifying barriers affecting dietary behaviour may contribute to individualized education and appropriate referral. However, it remains unclear whether existing food literacy assessment tools are suitable for nursing-related workflows, who should administer these tools, and how assessment findings can be translated into specific care actions within interdisciplinary healthcare systems.

Within the discipline of food literacy, there is a large variety of studies. The advantage of scoping reviews as a developing research technique is that it offers detailed overviews, which fill the existing gaps in the research and inform future investigations (28). Therefore, this scoping review aimed to synthesize adult food literacy assessment tools developed between 2015 and 2025, with particular attention to their dimensional evolution, cross-cultural adaptation strategies, and potential translation into community, clinical, nursing-related, and interdisciplinary care contexts, without assuming that food literacy assessment is a nurse-specific responsibility.

## 2 Materials and methods

This scoping review was conducted based on the methodological framework proposed by Arksey and O’Malley and reported in accordance with the Preferred Reporting Items for Systematic Reviews and Meta-Analyses Extension for Scoping Reviews (PRISMA-ScR) (28, 29). The review was registered on the Open Science Framework (OSF; Registration DOI: https://doi.org/10.17605/OSF.IO/3T6M5). The review was retrospectively registered on the Open Science Framework after literature screening had commenced. The registration documented the review objectives, eligibility criteria, and major methodological procedures and was intended to enhance transparency in the review process.

As this study synthesized previously published literature and did not involve direct recruitment of human participants or identifiable individual-level data, institutional ethical approval was not required.

### 2.1 Review questions and PCC framework

The review questions and eligibility criteria were developed according to the Population–Concept–Context (PCC) framework.

(1) Population: adults aged 18 years or older; (2) Concept: the core concept was food literacy assessment tools. This review focused on tools developed, validated, culturally adapted, localized, or applied for assessing food literacy among adults. Particular attention was given to evidence related to tool development processes, psychometric validation, cross-cultural adaptation procedures, and tool-related applications; (3) Context: the context was not restricted by country, region, or healthcare setting and included community, public health, primary care, clinical, and other adult health-related settings.

The primary review question was: What food literacy assessment tools for adults have been developed, validated, culturally adapted, or applied since 2015, and what are their measurement domains, cultural adaptation strategies, and practical applicability across different settings?

Four subquestions were addressed: (1) What food literacy assessment tools are currently available for adult populations across different countries and regions? (2) What dimensions and measurement domains are represented in these tools? (3) What methods and strategies have been used in the cross-cultural adaptation and validation of food literacy assessment tools? (4) What evidence exists regarding the use of these tools in community, clinical, and nursing-related practice, including who administers them and whether results inform subsequent actions?

### 2.2 Information sources and search strategy

A comprehensive literature search was conducted across seven electronic databases: PubMed, Web of Science, Embase, Wanfang Data, SinoMed, China National Knowledge Infrastructure (CNKI), and VIP Database. The search covered studies published from 1 January 2015 to 16 November 2025.

The lower date limit of January 2015 was selected on conceptual grounds. The seminal work by Vidgen and Gallegos, published in 2014, provided a foundational conceptualization and framework for food literacy that subsequently informed research on food literacy measurement. Therefore, the present review focused on the post-2014 period to map the development, validation, cross-cultural adaptation, and application of food literacy assessment tools following this conceptual consolidation.

The search strategy combined database-specific subject headings and free-text terms related to adult populations, food literacy, and assessment tools. Population-related terms included, where appropriate, “adult,” “middle-aged,” “aged,” “elderly,” and “older.” Concept-related terms included “food literacy” and related expressions, whereas instrument-related terms included “assessment,” “evaluation tool,” “scale,” “questionnaire,” and “assessment instrument.” Search terms and syntax were adapted to the indexing system and requirements of each database.

The search strategy was initially developed by a graduate student in nursing and subsequently reviewed and refined by the research team. In addition to electronic database searching, the reference lists of included studies and relevant articles were manually examined using backward citation searching to identify potentially eligible studies that might not have been retrieved through the database searches.

Grey literature was not included in this review. This decision was made because the present review focused on peer-reviewed empirical studies to ensure that included instruments had sufficient methodological information regarding their development, psychometric evaluation, cultural adaptation, or application characteristics. Specifically, dissertations and theses, conference abstracts, governmental or non-governmental organization toolkits, and other non-peer-reviewed instruments or practice materials were excluded.

This restriction was adopted because the review aimed to synthesize peer-reviewed empirical evidence containing sufficiently detailed information on instrument development, psychometric validation, cross-cultural adaptation, or documented implementation, thereby improving the consistency of data charting and comparison across tools. However, this restriction may have resulted in the omission of potentially relevant unpublished or non-peer-reviewed tools and should therefore be acknowledged as a limitation of the review. All retrieved records were imported into EndNote X9 for reference management and duplicate identification.

### 2.3 Eligibility criteria and study selection

Eligibility criteria were aligned with the PCC framework and the instrument-focused purpose of this review.

Studies were eligible for inclusion if they met all of the following criteria: (1) Participants were adults aged 18 years or older; (2) The study was an original empirical investigation published in a peer-reviewed journal; (3) The study examined an instrument explicitly developed, validated, culturally adapted, localized, or applied in a manner that generated instrument-relevant evidence for the assessment of food literacy as the primary construct; (4) The study reported information relevant to instrument development, psychometric properties, cross-cultural adaptation, scoring or interpretation, feasibility, administration, or implementation; (5) The article was published in Chinese or English between 1 January 2015 and 16 November 2025.

Food literacy is a multidimensional construct and may incorporate content related to nutrition knowledge, food selection, dietary behaviour, food preparation, food skills, and healthy eating. Therefore, overlap with these areas was not itself considered a reason for exclusion. Eligibility was instead determined according to the primary construct and conceptual purpose explicitly stated by the original instrument developers. Instruments designed exclusively to assess nutrition literacy, nutrition knowledge, dietary quality, nutritional status, food security, cooking skills, or other related constructs were excluded when food literacy was not explicitly defined as the primary target construct.

Studies applying an existing food literacy instrument were included only when they provided substantive information about the instrument itself, such as psychometric performance, feasibility, administration burden, scoring or interpretation, acceptability, cross-cultural performance, or implementation in practice. Validation studies were included when they reported psychometric evaluation or adaptation-related evidence of the instrument. Application studies were included only when the use of the instrument generated additional information regarding feasibility, interpretation, implementation characteristics, or practice translation. Intervention, cross-sectional, cohort, longitudinal, case–control, or other empirical studies that merely used a food literacy instrument as an outcome, predictor, or descriptive measure without providing additional instrument-relevant evidence were excluded.

Additional exclusion criteria were: (1) duplicate publications; (2) articles for which the full text could not be obtained; (3) publications in languages other than Chinese and English; (4) reviews, editorials, letters, books or book chapters, dissertations and theses, conference abstracts, and other non-peer-reviewed publications; (5) comprehensive health, dietary, or nutritional assessment instruments in which food literacy was not explicitly defined as the primary construct being assessed.

Duplicate records were identified using the duplicate-detection function in EndNote X9 and subsequently checked before screening. Two reviewers independently conducted the study-selection process. Title and abstract screening was performed sequentially against the predefined eligibility criteria. During the initial screening, records that were clearly unrelated to the review topic, published in languages other than Chinese or English, or classified as ineligible publication types were excluded. The titles and abstracts of the remaining records were then further assessed, with particular attention to the eligible population and whether the study provided evidence relevant to food literacy assessment tools. Reports considered potentially eligible after title and abstract screening were sought for full-text retrieval and subsequently assessed against the complete eligibility criteria. Disagreements between the two reviewers at any stage were resolved through discussion; when consensus could not be reached, a third reviewer was consulted for arbitration. The numbers of records excluded at each stage, reports that could not be retrieved, and studies included in the review were documented in the PRISMA-ScR flow diagram.

Because the focus of this review was the characteristics and application pathways of food literacy assessment tools rather than the effects of specific interventions or epidemiological associations, inclusion was not restricted according to conventional epidemiological study designs. Instead, eligibility was determined according to whether studies provided relevant evidence regarding instrument development, validation, cultural adaptation, feasibility, administration, interpretation, or implementation.

### 2.4 Data charting

Data were charted from the included studies according to the review questions and the characteristics relevant to food literacy assessment tools. For studies reporting newly developed instruments, the information charted included publication year, country or region, target population, instrument characteristics, assessment dimensions and content, number of items, completion time where reported, psychometric properties, and development or application settings. For studies involving cross-cultural validation or adaptation, additional information was charted on the original instrument, target population, adaptation context, translation and adaptation procedures, modifications made during adaptation, psychometric properties, and reported applications. To examine the potential translation of food literacy assessment into community and clinical practice, information on application settings, potential users, administration burden, scoring or domain interpretation, and any reported links between assessment results and subsequent education, intervention, referral, or health-management actions was also recorded. Nursing-related applicability was therefore explored as one potential implementation context rather than as a predefined eligibility criterion.

Two reviewers conducted the data-charting process and compared the extracted information. Discrepancies were discussed between the two reviewers, and when agreement could not be reached, a third researcher was consulted. The data-charting form was developed based on the review questions and was refined through discussion among the research team before formal data extraction.

### 2.5 Data synthesis

The charted data were synthesized descriptively in accordance with the objectives of the review. Basic characteristics of the included assessment instruments, including publication year, country or region, target population, instrument characteristics, assessment domains, and psychometric properties, were summarized and compared across studies.

To examine the dimensional characteristics of food literacy assessment, the dimensions and assessment content reported in the original studies were compared across instruments. Conceptually similar content was grouped to facilitate comparison, and the recurring domains represented across the instruments were subsequently summarized in relation to skills, knowledge, behaviours, and attitudes. This process was intended to describe similarities and differences in instrument content rather than to generate or validate a new measurement model.

For cross-culturally adapted or validated instruments, adaptation-related information reported in each included study was extracted and synthesized. Specifically, two reviewers independently reviewed the full texts of included studies and identified all reported cultural adaptation procedures, including translation processes, item modifications, changes in measurement structures, adjustments to dietary references or guidelines, and other context-specific modifications. Each reported adaptation procedure was coded according to its primary purpose and the type of modification involved.

Through iterative comparison and discussion, conceptually similar adaptation procedures were grouped into broader categories. The initial categories were inductively derived from the characteristics of adaptation strategies reported in the included studies rather than predefined from an existing framework. The two reviewers independently compared the coded adaptation strategies and discussed discrepancies until consensus was reached. When disagreement remained, a third reviewer was consulted for final resolution.

Based on this inductive process, adaptation strategies were descriptively categorized into three groups: language and semantic adaptation, structural and measurement adaptation, and conceptual and value embedding. Language and semantic adaptation included translation, back-translation, wording modification, and adjustments aimed at improving linguistic equivalence. Structural and measurement adaptation referred to modifications involving items, dimensions, factor structures, response formats, or other measurement characteristics. Conceptual and value embedding referred to adaptations incorporating culturally specific dietary practices, dietary guidelines, beliefs, values, or contextual meanings. These categories were used only as a descriptive framework to organize and summarize adaptation practices reported in the included studies and were not intended to represent a validated theoretical model of cultural adaptation.

In this review, cross-cultural adaptation was not considered synonymous with achieving measurement equivalence alone. Instead, adaptation procedures were interpreted as approaches aimed at maintaining the core construct of food literacy while improving cultural relevance and contextual applicability in different populations.

Finally, evidence relating to the practical application of the instruments was summarized descriptively, with particular attention to the settings in which instruments had been used, potential users, administration burden, interpretation of assessment results, and whether assessment findings were linked to subsequent education, intervention, referral, or health-management actions. This synthesis was used to identify current patterns and gaps in the translation of food literacy assessment into community, clinical, and nursing practice.

## 3 Results

### 3.1 Search results

After duplicate removal, 192 records remained for title and abstract screening. During the initial screening, 107 records were excluded, including 7 records published in languages other than Chinese or English, 96 records that were not relevant to the review topic or eligibility criteria, and 11 records categorized as other types of literacy. The titles and abstracts of the remaining 85 records were further screened against the predefined eligibility criteria. Of these, 47 records were excluded because the study population did not meet the eligibility criteria (*n* = 33) or because the study did not meet the instrument-related eligibility criteria (*n* = 14). Thirty-eight reports were subsequently sought for full-text retrieval. Ten reports could not be retrieved, leaving 28 reports for full-text eligibility assessment, all of which were included in the final review. The study-selection process is shown in Figure 1.

**Figure 1:**
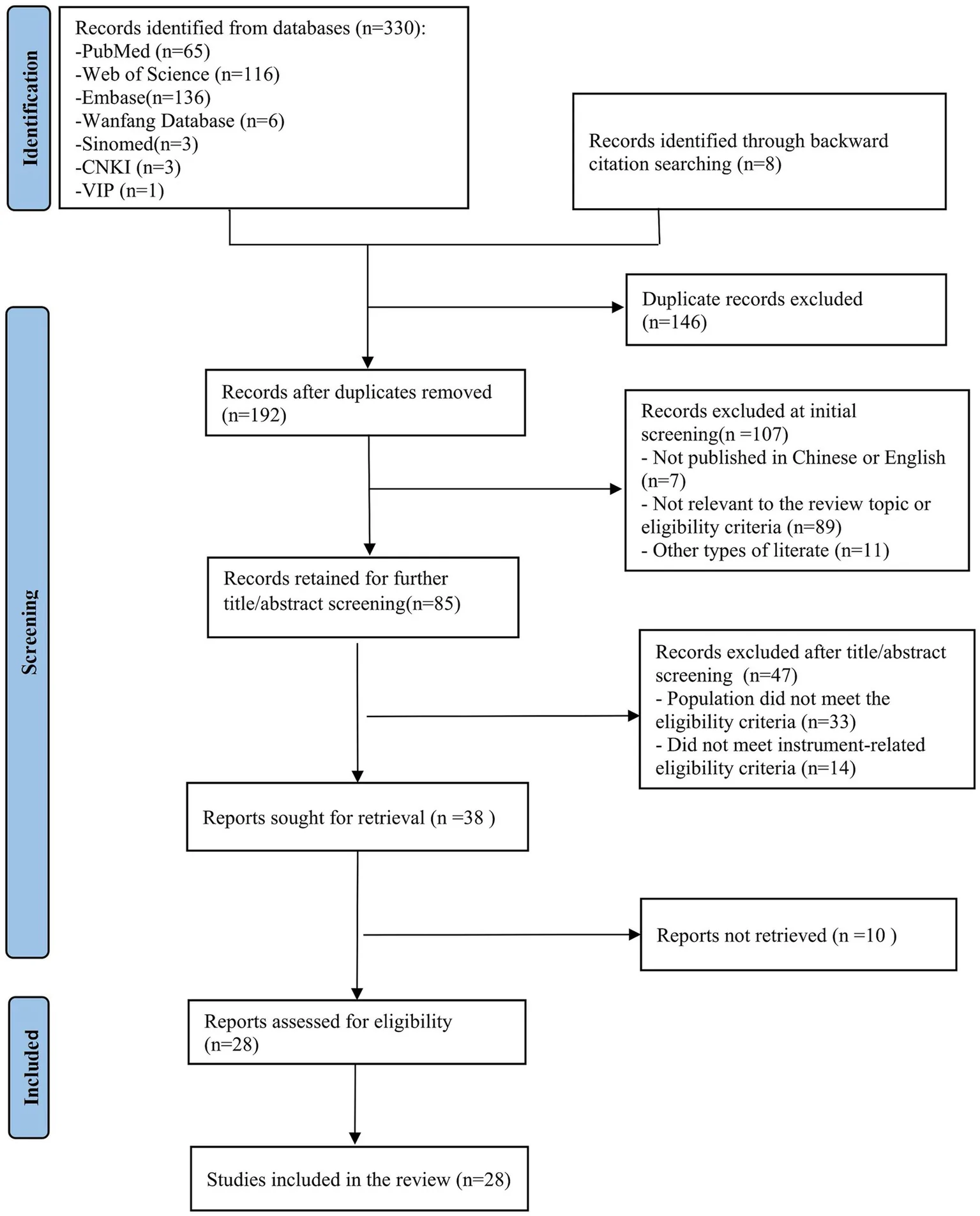
Literature screening process and results.

### 3.2 General characteristics of assessment tools

A total of 28 studies involving 21 adult food literacy assessment tools were included in this review, comprising 13 newly developed instruments and eight culturally adapted or validated instruments (Tables 1, 2).

**Table 1:** Developed food literacy assessment tools.

Tool name	Year & country	Population	Dimensions	Core domains	Practical application characteristics
Food Literacy Behaviour Checklist (30)	2018-Australia	Low- and middle-income adults	① Planning and management; ② Selection; ③ Preparation	① Skills; ② Behaviour	Advantages: ① Highly targeted: Meets the requirements of community nutrition education programs; ② Can quickly assess changes in dietary behaviours and guide adjustments to community intervention strategies; Limitations: ① Lack of validation in clinical populations; ② Single-dimensional, making it difficult to comprehensively reflect the psychological and social factors, such as cognition and attitude, required in clinical settings.
SPFL (8)	2018-Netherlands	Adults	① Food preparation skills; ② Resilience and resistance; ③ Healthy snacking; ④ Social and mindful eating; ⑤ Food label checking; ⑥ Dietary planning; ⑦ Healthy budget management; ⑧ Food stocking	① Skills; ② Behaviour; ③ Attitude	Advantages: Clear structure, good reliability and validity, and capable of quantifying food literacy levels and predicting healthy eating behaviours; Limitations: ① Lack of validation in clinical and community populations; ② The scale relies on self-perception, which may introduce social desirability bias. Accuracy can be improved by combining it with objective dietary records.
SFLQ (31)	2018-Switzerland	Adults	① Functional food literacy; ② Interactive food literacy; ③ Critical food literacy	① Skills; ② Knowledge	Advantages: Concise and efficient: The entries are concise and suitable for large-scale community surveys or rapid clinical evaluations; Limitations: ① Dependent on subjective scoring, thus it is recommended to combine with objective indicators; ② Predominantly for adults with high educational attainment, its applicability needs further verification; ③ Not validated in community or clinical settings
Food literacy measurement tool for young Korean adults (33)	2020-South Korea	Adults	1. Knowledge areas: ① Food and nutrition knowledge; ② Food safety; ③ Food system; ④ Social and cultural background; 2. Skill areas: ① Food skills; ② Self-efficacy; ③ Food selection; ④ Food resource management	① Skills; ② Behaviour; ③ Attitude; ④ Knowledge	Advantages: ① Comprehensive content, suitable for assessing the influencing factors of complex dietary behaviours; ② Applicable for evaluating food literacy among young people; Limitations: ① The items are too long, which hinders their promotion in large-scale communities or clinical settings; ② In terms of applicability, it was only developed for young adults in South Korea, and culturally specific items may limit its direct application in other populations, requiring local adaptation; ③ It has not been validated in community or clinical settings.
EFLBQ (38)	2020-USA	University students	① Health and nutrition; ② Flavor; ③ Food preparation; ④ Planning and decision-making; ⑤ Convenience	①Skills	Advantages: Focuses on the behavioural aspect, making it easier to guide practical applications compared to traditional knowledge-based questionnaires; Limitations: Limited to college students and does not cover the general population in the community or clinical patients.
Comprehensive Food Literacy Measurement Tool (34)	2020-South Korea	Adults	① Preparation and cooking; ② Production; ③ Selection; ④ Disposal; ⑤ Intake	① Skills; ② Behaviour; ③ Attitude	Advantages: Covers the entire process from production to consumption, with strong environmental awareness; Limitations: ① The sample is not representative enough and needs to be tested in other adult populations; ② Some dimensions have high requirements for participants and investigators; ③ It does not involve validation in community or clinical patient groups.
FLitT (39)	2021-USA	Low-income adults	① Knowledge; ② Self-efficacy; ③ Behaviour; ④ Consumption frequency	① Skills; ② Behaviour; ③ Attitude; ④ Knowledge	Advantages: ① Highly targeted: Can be directly used for nutritional intervention assessment; ② Simple implementation: The reading difficulty is suitable for groups with low educational levels; Limitations: ① Too many items hinder its promotion in large-scale communities; ② Not verified in community or hospital settings.
Food Literacy Assessment Tool for Community-Dwelling Elderly People (35)	2021-South Korea	Community elderly	① Preparation and cooking; ② Allocation; ③ Production and disposal	① Skills; ② Behaviour; ③ Attitude; ④ Knowledge	Advantages: ① Simplicity and feasibility: The questionnaire is short in length and suits the cognitive characteristics of the elderly and the need for rapid assessment; ② Targeted: Specifically developed for community-dwelling elderly, it has high reference value for food nutrition intervention programs for this group; Limitations: ① Insufficient sample representativeness: The majority of participants are female and have low educational levels. The applicability in other groups needs further verification; ② Not tested clinically.
FOODLIT-tool (32)	2022-Portugal	Adults	① Cooking skills; ② Selection and planning; ③ Production and quality; ④ Environmental safety; ⑤ Source traceability	① Skills; ② Attitude; ③ Knowledge	Advantages: Multi-dimensional assessment: It can comprehensively evaluate an individual’s performance in food-related knowledge, skills, and behaviours; Limitations: Insufficient sample representativeness: Further validation is needed in clinical settings.
Food Literacy Questionnaire for Diabetic Patients (36)	2022-Iran	Adults with diabetes	① Food facts reading ability; ② Food classification practical ability; ③ Food calorie calculation ability; ④ Understanding of food’s health impact; ⑤ Ability to prepare a healthy diet	① Skills; ② Knowledge	Advantages: ① Targeted: Specifically designed for diabetic patients, covering the special needs in disease management; ② Practicality: Identifies high-risk individuals through assessing their food literacy levels, providing targeted goals for nutritional intervention; Limitations: ① Many items, making it difficult to implement in busy clinical settings and large-scale community screenings; ② The samples are from community centers and have not been verified clinically.
FNLQ (37)	2022-China	Adults	① Knowledge and understanding; ② Acquisition and selection ability; ③ Ability to prepare food; ④ Eating habits and behaviours	① Skills; ② Behaviour; ③ Knowledge	Advantages: Integrates Chinese dietary culture and has good universality in both urban and rural areas, making it suitable for national-level nutritional health surveys; Limitations: ① Uncertain threshold of nutritional literacy: Lack of dietary intake data, unable to determine the optimal threshold of nutritional literacy through dietary quality index, only set subjectively, and the scientificity needs to be verified; ② Not tested in communities or clinical settings.
East African Food Literacy Scale (41)	2024—Uganda & Kenya	Adults	① Planning and management; ② Selecting ability; ③ Preparation ability; ④ Practical consumption; ⑤ Information evaluation	① Skills; ② Behaviour; ③ Attitude; ④ Knowledge	Advantages: Cultural sensitivity: Developed in the context of limited resources such as “food deserts,” it has strong applicability for public health projects in East African countries; Limitations: ① Insufficient sample representativeness: Most participants have higher education and are employed, which may not represent those with lower educational levels or the unemployed, limiting the universality of the results; ② Its validation is mainly based on community populations, which may limit its direct application in clinical settings.
CFLM (40)	2024-Canada	Adults	① Nutritional literacy; ② Agricultural food system literacy; ③ Media literacy; ④ Cultural literacy; ⑤ Civic literacy; ⑥ Ecological literacy	① Skills; ② Attitude; ③ Knowledge	Advantages: ① Multi-dimensional coverage: It can comprehensively assess food-related knowledge and skills, providing baseline data for intervention; ② Forward-looking: It covers civic and ecological literacy, with strong comprehensiveness; Limitations: ① Insufficient sample size: Only 154 participants were included; ② Bias in population representation: The sample lacks representativeness regarding gender, age, and region, affecting generalizability; ③ No community or clinical validation was conducted

SPFL, Self-Perceived Food Literacy Scale; SFLQ, Short Food Literacy Questionnaire; EFLBQ, Eating and Food Literacy Behaviours Questionnaire; EFLBQ, Eating and Food Literacy Behaviours Questionnaire; FLitT, Food Literacy Assessment Tool; FOODLIT-tool, the adaptable food literacy Tool; FNLQ, Food and Nutrition Literacy Questionnaire; CFLM, Canadian Food Literacy Measure.

**Table 2:** Cross-culturally validated food literacy assessment tools.

Tool name	Year/development and validation	Cultural adaptation method	Cultural adaptation strategy	Measurement characteristics
FLEQ-Ch (42)	2020-Development: Australia Verification: Chinese residents	① Translation and back-translation; ② Delphi expert consultation; ③ Pre-survey	1. Language and semantic adaptation: A translation-back-translation process was carried out to eliminate the ambiguities caused by direct translation and to clearly define the operationalized definition of “healthy food” in accordance with Chinese food safety standards; 2. Structure and measurement adaptation: ① Item addition and deletion: New items aligned with local dietary culture were added; ② Dimension reconfiguration: The “preparation” dimension was focused on “cooking attitude” to align with Chinese family cooking culture; 3. Concept and value embedding: Used stratified sampling of urban and rural areas to ensure the universality of the tool in Chinese environments; and adjusted items to reflect integration of local dietary concepts and food safety practices.	1. Reliability is good: Cronbach’s *α* = 0.869, ICC = 0.948; 2. Validity is good: I-CVI = 0.560–1.0, CVI = 0.945; CFA shows good model fit.
SFLQ-Br (43)	2022-Development: Switzerland Verification: Brazilian adults	① Conceptual and item equivalence; ② Semantic equivalence; ③ Operational equivalence; ④ Measurement equivalence	1. Language and semantic adaptation: Simplified complex expressions and removed vague temporal terms to avoid recall bias; 2. Concept and value embedding: Replace the “Swiss Food Pyramid” in the original scale with the official Brazilian guideline “Dietary Guidelines for the Brazilian Population,” and adjust the relevant items.	1. Reliability is good: McDonald’s *ω* = 0.874, and the Kappa values are all greater than 0.500; 2. Validity is good: S-CVI = 0.930.
PL-SFLQ (46)	2022-Development: Switzerland Verification: Polish adults	① Forward translation; ② Expert group consensus; ③ Reverse translation; ④ Pre-test	1. Language and semantic adaptation: Used clear Polish expressions; Included both “Healthy Nutritional Pyramid” and “Healthy Nutritional Plate” in the entries; 2. Structure and measurement adaptation: EFA and CFA confirmed a three-factor model more suitable for the Polish sample than the original single-factor model; 3. Concept and value embedding: Ensure that the content of the scale is in sync with the latest national nutrition guidelines and policies of Poland.	1. Reliability is good: Cronbach’s *α* = 0.841 2. Structural validity is good: The three-factor model fits better than the single-factor model of the original scale
the Spanish adaptation of the SPFL scale (47)	2022-Development: The Netherlands Verification: Spanish university students	① Translation and back-translation; ② EFA and CFA; ③ Item adjustment	1. Language and semantic adaptation: Translation and back-translation were conducted; 2. Structure and measurement adaptation: ① Dimension reconfiguration: Factor analysis adjusted the original seven-factor model to a five-factor model, better suited for the Spanish university student population; ② Item deletion: Items inconsistent with the cultural background were eliminated.	1. Reliability is good: Cronbach’s *α* = 0.894; 2. Validity is good: The fit index of the five-factor model is superior to that of the original seven-factor model.
The Turkish version of the FOODLIT-Tool (44)	2024-Development: Spain Verification: Turkish adults	① Translation and back-translation ② Expert review; ③ Pre-survey	1. Language and semantic adaptation: Adjust the wording of the items based on the feedback from the pre-survey to ensure that the language is easy to understand; 2. Structure and measurement adaptation: Verified and retained the five-factor structure and 24 items of the original scale to ensure the cross-cultural consistency of its theoretical framework in the Turkish context; 3. Concept and value embedding: Calculate the CVI through expert scoring to ensure item consistency with Turkish cultural concepts of food literacy.	1. Reliability is good: The Cronbach’s α coefficient is 0.927; 2. Validity is good: CFA shows that the model fits well.
SPFL-SF (49)	2024-Development: The Netherlands Verification: Austrian adults	① Language and context adjustment; ② Content modification; ③ Expert consultation	1. Language and semantics adaptation: Adjusted the language and context; 2. Structure and measurement adaptation: Refined original dimensions into 10 factors using EFA, simplifying and reducing items to 20.	1. Reliability is good: Cronbach’s *α* = 0.770, McDonald’s *ω* = 0.660–0.890; 2. Validity is good: 10-factor model, the data model fits excellently (SRMSR = 0.020, RMSEA = 0.020, CFI = 1.000, TLI = 1.000)
SFLQ-HU (48)	2025-Development: Switzerland Verification: Hungarian university students	① Translation; ② Synthesis; ③ Back-translation; ④ Pre-test; ⑤ Internal consistency assessment; ⑥ External convergent validity test	1. Language and semantic adaptation: An interdisciplinary team ensured the scale conformed to the Hungarian language and cultural context; 2. Structure and measurement adaptation: Based on the results of the pre-test, the presentation was optimized to ensure the comprehensibility of the items among the target population; 3. Concept and value embedding: Replace “Swiss Food Pyramid” with the latest Hungarian nutrition recommendation “OKOSTÁNYÉR®,” and adjust “Swiss official recommendation” to “Hungarian official recommendation”	1. Reliability is good: Cronbach’s *α* = 0.850, ICC = 0.921; 2. Validity is good: CFA supports a unidimensional structure, and the model fits well.
The Arabic version of the SFLQ (45)	2025-Development: Switzerland Verification: Lebanese adults	① Use the two-way translation method; ② Expert review; ③ Pre-test	1. Language and semantic adaptation: Through translation and expert review, ensure that the language expression conforms to Arabic language conventions; 2. Structure and measurement adaptation: Through multiple sets of CFA, test the measurement invariance between genders to ensure the consistency of the scale structure in different gender groups; 3. Concept and value embedding: While retaining the core construct of the original scale, ensure that the expression of all items is in line with the Lebanese dietary cultural background.	1. Reliability is good: McDonald’s *ω* = 0.860, Cronbach’s *α* = 0.860; 2. Structural validity is good: The single-factor model after modification fits well.

SPFL-SF, Self-Perceived Food Literacy Scale Short Form; FLEQ-Ch, Food Literacy Evaluation Questionnaire Chinese Version; PL-SFLQ, Short Food Literacy Questionnaire for Polish; SFLQ-Br, Short Food Literacy Questionnaire for Brazil; SFLQ-HU, Short Food Literacy Questionnaire for Hungarian.

Newly developed instruments were defined as tools for which the included studies reported original item generation, domain construction, and scoring procedures. Culturally adapted or validated instruments referred to previously established tools that underwent translation, cultural modification, or psychometric evaluation in a different population or context. The characteristics of the included tools were summarized according to publication trends, geographic distribution, target populations, and development pathways.

The included studies reporting these tools were mainly published between 2018 and 2025, with an increased number of studies reported after 2020. These instruments were developed across multiple regions, including Oceania (Australia) (30), Europe (the Netherlands, Switzerland, and Portugal) (8, 31, 32), Asia (South Korea, China, and Iran) (33–37), North America (the United States and Canada) (38–40), and Africa (Uganda and Kenya) (41). Among the 13 newly developed instruments, tools originated from multiple geographical regions, including Oceania, Europe, Asia, North America, and Africa (8, 30–35, 38–40). These geographical distributions reflected variation in cultural and dietary contexts rather than a predefined economic classification.

Regarding target populations, earlier studies generally focused on broad adult populations, whereas more recent instruments increasingly targeted specific groups, including university students, community-dwelling older adults, low- to middle-income adults, and adults with type 2 diabetes (27, 33, 35, 36, 39). This population-specific development was observed in several recently developed tools, such as instruments designed for older adults, individuals with diabetes, and adults experiencing socioeconomic disadvantages.

Two major development pathways were identified among the included instruments. The first pathway involved the development of new assessment tools based on different conceptual frameworks of food literacy. These instruments incorporated various domains, including skills, knowledge, behaviours, and attitudes, with some extending assessment content to food systems and sustainability (8, 31, 32). The second pathway involved cross-cultural adaptation and validation of existing instruments. Eight instruments originally developed in countries such as Australia and Switzerland were subsequently adapted and validated in other cultural contexts, including China, Brazil, Turkey, Poland, Hungary, and Lebanon (42–44).

### 3.3 Dimensional focus of the assessment tools

To compare the dimensional characteristics of the included food literacy assessment tools, the reported assessment domains and item contents were extracted from each original study. Conceptually similar items were grouped according to the four core domains proposed by Cullen et al., including skills, knowledge, behaviours, and attitudes. Each newly developed instrument was subsequently mapped according to whether these domains were represented in its assessment framework. Domain classification was based on the conceptual descriptions provided in the original instrument development studies.

Among the 13 newly developed food literacy assessment tools, all tools included skill-related components. These components primarily encompass practical capabilities related to food preparation, meal planning, food selection, budget allocation, and resource management. Knowledge-related components are incorporated in 9 out of the 13 tools, accounting for 69.2%, including the Short Food Literacy Questionnaire (SFLQ), Food literacy measurement tool for young Korean adults, Food Literacy Assessment Tool (FLitT), Food Literacy Assessment Tool for Community-Dwelling Elderly People, the adaptable food literacy Tool (FOODLIT-tool), Food Literacy Questionnaire for Diabetic Patients, Food and Nutrition Literacy Questionnaire (FNLQ), East African Food Literacy Scale and Canadian Food Literacy Measure (CFLM) (31, 32, 34–37, 39–41). The knowledge-related components cover domains including nutritional knowledge, food label interpretation, food safety, awareness of food-related information, and comprehension of the association between dietary choices and health outcomes.

Behaviour-related components were incorporated into instruments through assessment of dietary habits, food consumption patterns, meal preparation behaviours, and daily food-related practices. Attitude-related components were included in 8 of the 13 tools (61.5%), with assessed contents including self-efficacy, confidence in food preparation, perceptions of food choices, environmental awareness, and cultural considerations related to eating behaviours. The remaining (38.5%) did not include this area (7, 25, 32). These instruments included the Self-Perceived Food Literacy Scale (SPFL), Food Literacy Measurement Tool for young Korean adults, Comprehensive Food Literacy Measurement Tool, FLitT, Food Literacy Assessment Tool for Community-Dwelling Elderly People, FOODLIT-tool, East African Food Literacy Scale and CFLM.

Several instruments incorporated broader domains beyond individual dietary competencies. Some tools developed after 2020 incorporated assessment contents related to food systems, sustainability, environmental impacts, or social aspects of food choices. These instruments included SPFL, Food Literacy Measurement Tool for Young Korean Adults, CFLM, FLitT, Food Literacy Assessment Tool for Community-Dwelling Elderly People, FOODLIT-tool, East African Food Literacy Scale, and Canadian Food Literacy Measure (8, 32–35, 39–41).

Overall, the included instruments differed in the number and combination of assessed domains. While skills-related components were consistently represented across all newly developed tools, knowledge, behaviour, and attitude domains varied in their coverage and specific assessment contents among instruments.

### 3.4 Applicability of assessment tools in community and clinical practice

The practical applicability of food literacy assessment tools was examined according to their intended settings, target populations, number of items, completion time, and reported use characteristics. These factors may influence its practicality in community and clinical settings, as well as the various correlations between measurement depth and implementation speed.

In community practice, assessment tools must meet the requirements of rapid screening, large-scale administration, and program evaluations for nutrition education. Community-oriented tools generally emphasize shorter completion time and simplified items to facilitate screening and nutrition education programs. As shown in Table 1, tools like the Food Literacy Behaviour Checklist and SFLQ are especially appropriate for baseline surveys in community nutrition programs (30, 31). As well, tools specifically designed for the community-dwelling elderly, such as the Food Literacy Assessment Tool for Community-Dwelling Elderly People (35), employ simplified item statements tailored for elderly populations, clearly targeting community nutritional programs. However, such tools may lack depth, feature limited dimensional coverage, and require further validation within community or clinical settings, particularly concerning psychosocial factors. As a result, despite having significant potential as quick screening tools, their current depth and generalizability are still restricted. Also, by directly connecting assessment content to resource-constrained food choices and decision-making, like the Food Literacy Behaviour Checklist and FLitT example explicitly target low-income individuals and provide information that may support targeted nutrition education or intervention planning (30, 39).

In clinical settings, instruments designed for specific health conditions were identified among the included studies. Because of this, these tools require extensive content depth, disease-specific focus, and psychometric resilience. Several of the offered tools are tailored to certain clinical populations. For instance, the Food Literacy Questionnaire for Diabetic Patients is designed specifically for adult diabetic patients and addresses topics such as understanding dietary patterns, the relationship between nutrition and blood sugar, and portion control, all of which are related to disease management (36). However, explicit evidence linking assessment scores to subsequent nursing interventions or referral pathways was rarely reported. Besides, the number of items differs noticeably when assessment tools are compared. Some, such as the Food Literacy Measurement Tool for Young Korean Adults (33), span broad areas (“skills,” “knowledge,” “behaviour,” and “attitude”) with more items and longer completion times, but others are more brief. Such characteristics may limit their routine use in time-constrained clinical environments.

The evidence directly demonstrating the use of food literacy assessment tools administered by nurses or integrated into routine nursing care workflows is limited in the included studies. Some tools have been developed or applied in community-oriented settings where nurses may participate in screening, health education, or follow-up, such as the Food Literacy Behaviour Checklist, the Food Literacy Assessment Tool for Community-Dwelling Elderly People, and the Food Literacy Questionnaire for Diabetic Patients (30, 35, 36). However, the included studies did not consistently report nurses as the explicit administrators of these tools, nor did they provide detailed descriptions of implementation procedures specifically designed for nursing workflows.

Overall, the included tools varied in their intended settings, assessment depth, and implementation characteristics. Differences were observed between brief screening-oriented instruments and more comprehensive tools designed for detailed assessment.

### 3.5 Cultural adaptation strategies of assessment tools

Eight assessment tools underwent cross-cultural adaptation and validation across different populations. As shown in Table 2, although the specific procedures varied among studies, the reported adaptation strategies could be descriptively summarized into three recurring categories: language and semantic adaptation, structure and measurement adaptation, and concept and value embedding.

Language and semantic adaptation were reported for all eight tools. Common procedures included forward translation, back-translation, expert review, consensus procedures, and pre-testing. In addition to achieving linguistic equivalence, several studies modified wording or expressions to improve comprehensibility and relevance in the target population. For example, the Food Literacy Evaluation Questionnaire Chinese Version (FLEQ-Ch) (42) clarified the operational definition of “healthy food” according to Chinese food safety standards, while the Short Food Literacy Questionnaire for Brazil (SFLQ-Br) (41) simplified complex expressions and removed vague temporal terms. The Turkish version of the FOODLIT-Tool (44) and the Arabic version of the SFLQ (45) also adjusted item wording to improve linguistic and cultural appropriateness in their respective target populations.

Structure and measurement adaptation was explicitly reported for seven of the eight tools. The FLEQ-Ch (42) involved item addition and deletion and reconfigured the “preparation” dimension to emphasize cooking attitudes in the Chinese context. The Short Food Literacy Questionnaire for Polish (PL-SFLQ) (46) was reorganized from the original single-factor structure into a three-factor model, whereas the Spanish adaptation of the SPFL scale (47) was adjusted from a seven-factor to a five-factor structure. The Self-Perceived Food Literacy Scale Short Form (SPFL-SF) (46) was further refined into a 10-factor, 20-item version. In contrast, the Turkish version of the FOODLIT-Tool (44) retained the original five-factor, 24-item structure, while the Short Food Literacy Questionnaire for Hungarian (SFLQ-HU) (47) and the Arabic version of the SFLQ (45) evaluated the suitability and stability of their measurement structures in the target populations. The SFLQ-Br (43) did not explicitly report a separate structure and measurement adaptation strategy in Table 2.

Concept and value embedding was reported for six tools. This strategy involved aligning the content of the adapted instrument with local dietary concepts, national dietary recommendations, food-related practices, or other culturally specific contexts. For example, the FLEQ-Ch incorporated items reflecting Chinese dietary culture and food safety practices (42). The SFLQ-Br replaced the “Swiss Food Pyramid” with the “Dietary Guidelines for the Brazilian Population”, while the PL-SFLQ aligned its content with current Polish nutrition guidelines and policies (43, 46). Similarly, the SFLQ-HU replaced the Swiss dietary reference with the Hungarian “OKOSTÁNYÉR®” recommendation. Conceptual and cultural relevance was also explicitly considered in the Turkish version of the FOODLIT-Tool and the Arabic version of the SFLQ (44, 45, 48).

Overall, these tools, including FLEQ-Ch, PL-SFLQ, the Turkish version of FOODLIT-Tool, SFLQ-HU, and the Arabic version of SFLQ, have reported the adaptation strategies corresponding to all three categories (42, 44–46, 48). The SFLQ-Br incorporated language and semantic adaptation together with concept and value embedding but did not explicitly report structure and measurement adaptation as a separate strategy (43). In contrast, the Spanish adaptation of the SPFL scale (47) and the SPFL-SF (49) reported language and semantic adaptation and structure and measurement adaptation, but did not explicitly report concept and value embedding. Thus, the included studies differed in the breadth and emphasis of the cultural adaptation procedures applied.

These three categories were used solely to organize and describe recurring adaptation strategies reported in the included studies and should be interpreted as a descriptive analytical framework developed for synthesizing cultural adaptation strategies identified in the included studies.

## 4 Discussion

This scoping review identified an evolving pattern in adult food literacy assessment, with instruments expanding from individual food-related competencies toward broader contextual and system-oriented dimensions. Across the included instruments, skills-related components were consistently represented, covering practical capabilities such as food preparation, meal planning, food selection, budgeting, and resource management. This pattern is consistent with the conceptualization of food literacy proposed by Vidgen and Gallegos, which emphasizes the translation of knowledge and skills into food-related behaviours (7). However, the coverage of knowledge, behavioural, and attitudinal domains varied across instruments, and several newer tools incorporated broader considerations related to food systems, sustainability, and social contexts. These findings suggest that current food literacy assessment is gradually moving beyond the measurement of isolated individual capabilities toward a more contextualized understanding of how food-related competencies are applied in everyday life.

Beyond traditional nutrition-related knowledge, recent instruments have increasingly incorporated information-processing abilities within the knowledge domain. For example, the East African Food Literacy Scale and CFLM include elements related to media literacy and the evaluation of nutrition-related information, whereas other tools continue to focus primarily on knowledge of dietary recommendations (40, 41). This indicates that the knowledge dimension of food literacy is expanding from the acquisition of factual information toward the ability to interpret, evaluate, and apply food-related information in complex everyday environments. Future assessment development may therefore consider broader information-processing abilities when evaluating food literacy in populations exposed to increasingly diverse food information sources (50).

In addition to individual competencies, several recently developed instruments have incorporated system-oriented dimensions, including critical thinking, environmental awareness, and understanding of food systems (51, 52). For example, the CFLM extends assessment beyond individual behaviours by including civic and ecological literacy related to community food accessibility, environmental impacts of food packaging, and food waste reduction (40). These developments indicate that food literacy assessment is increasingly recognizing the influence of broader social and environmental contexts on food-related behaviours.

Another notable trend was the increasing development of population-specific instruments. Recent tools have been designed for groups such as university students, community-dwelling older adults, low-income adults, and individuals with type 2 diabetes, reflecting growing recognition that food literacy is shaped by population-specific needs and lived experiences. For example, instruments developed for individuals with diabetes have incorporated domains related to dietary patterns, nutrition–disease relationships, and portion control, reflecting considerations relevant to disease-specific dietary management (36).

Nevertheless, although recent instruments such as the EFLBQ and the Food Literacy Assessment Tool for Community-Dwelling Elderly People were developed for specific populations, the extent to which these tools capture variations in cultural backgrounds, socioeconomic conditions, and individual circumstances within these groups remains uncertain (35, 38, 53). Current instruments have largely contributed to population-specific assessment; however, many continue to focus primarily on measuring individual competencies rather than examining how these competencies are applied within everyday contexts. Future development of food literacy assessment tools may therefore benefit from greater consideration of contextual influences, including practical constraints in daily life, emotional factors, and environmental conditions that may shape food-related behaviours (54, 55). Although population-specific tools commonly assess relevant capabilities such as nutrition label interpretation, cooking skills, and food preparation practices, they often provide limited information regarding whether individuals can apply these capabilities, adapt them to changing circumstances, or overcome contextual barriers in their daily environments.

This limitation is particularly relevant for instruments developed for populations with distinct social and health contexts, such as community-dwelling older adults, university students, and individuals experiencing socioeconomic disadvantage. Among older adults, food literacy may involve not only cooking skills and food management knowledge but also contextual factors such as social support, access to information, and functional limitations that influence everyday food practices (56–58). Similarly, food literacy among university students may be shaped by factors including campus food environments, peer influences, and lifestyle constraints, which are not consistently incorporated into existing instruments (59, 60). For populations experiencing socioeconomic disadvantage, tools such as the Food Literacy Behaviour Checklist and FLitT have assessed skills related to budgeting and food choices; however, broader structural constraints, including food availability and time-related limitations, may require further consideration (54–58, 61). Therefore, while population-specific instruments improve relevance to particular groups, additional attention is needed to understand how food-related capabilities interact with social and environmental contexts.

These considerations are also important for translating food literacy assessment into healthcare and community settings. In nursing-related contexts, assessment tools may provide supportive information regarding individuals’ food-related capabilities and potential barriers but should not replace nutritional assessment or professional dietary management. Registered dietitians and clinical nutrition professionals remain central to comprehensive nutrition assessment and individualized dietary therapy, whereas nurses may contribute through patient education, care coordination, follow-up, and identification of barriers affecting the implementation of dietary recommendations (53, 62, 63). Therefore, food literacy assessment may support nurses and other healthcare professionals in identifying educational needs, facilitating appropriate referrals, and coordinating multidisciplinary care rather than serving as a nurse-specific responsibility (64, 65).

However, the included studies provided limited evidence regarding the direct use of food literacy assessment results to guide formal nursing diagnoses or intervention pathways. Future implementation research should clarify how assessment findings can be interpreted and translated into context-sensitive actions through collaboration among nurses, dietitians, physicians, public health professionals, and community health workers. In this context, food literacy assessment should be considered a complementary tool that strengthens assessment-to-education, referral, and follow-up processes within interdisciplinary care.

Cultural adaptation is an important consideration when applying food literacy assessment tools across different populations. In this review, eight cross-culturally adapted instruments were identified, and reported adaptation strategies were descriptively grouped into three categories: language and semantic adaptation, structural and measurement adaptation, and conceptual and value embedding. Among the included studies, translation, back-translation, expert review, and psychometric evaluation were the most frequently reported approaches. These findings indicate that current adaptation practices have largely focused on maintaining linguistic clarity and measurement consistency between original and adapted instruments. However, fewer studies have examined whether adapted tools adequately reflect culturally specific meanings, food practices, and contextual factors influencing food-related behaviours (66–68).

The limited reporting of conceptual and value embedding highlights the need to consider cultural relevance beyond direct translation. For example, the SFLQ-Br replaced the Swiss Food Pyramid with Brazil’s Official Dietary Guidelines, while the FLEQ-Ch incorporated aspects related to Chinese dietary culture and food safety practices (42, 43). These adaptations demonstrate the importance of balancing preservation of the core food literacy construct with the inclusion of locally meaningful food-related experiences. Therefore, cross-cultural adaptation should not be understood solely as achieving measurement equivalence, but also as ensuring conceptual relevance within specific cultural and social contexts.

Although some instruments incorporated culturally relevant modifications, these approaches were reported less frequently than linguistic and psychometric adaptations. Existing adaptations often focused on modifying dietary guidelines or adding culturally specific items, whereas fewer studies explored how food literacy is understood and experienced within different cultural settings. Furthermore, among the newly developed tools included in this review, instruments were mainly reported from Australia, Europe, North America, and selected Asian settings, indicating that further research is needed to examine food literacy constructs across a broader range of sociocultural environments. Future research should therefore move beyond translation and measurement adaptation toward greater consideration of cultural construct adaptation. Culturally grounded approaches, including qualitative and participatory methods, may help identify locally meaningful dimensions of food literacy while preserving the core construct across diverse populations and settings (69, 70).

Another challenge concerns the balance between assessment depth and implementation feasibility. Comprehensive instruments, such as the Food Literacy Measurement Tool for Young Korean Adults, provide multidimensional information but may require greater administration time, which may limit their routine use in time-constrained healthcare or community settings (33). In contrast, brief tools such as the Food Literacy Behaviour Checklist may be easier to implement but may provide less information regarding the broader psychosocial and contextual determinants influencing dietary behaviours (30). This challenge is consistent with previous evidence indicating that limited time, training needs, and competing clinical priorities can influence the delivery of nutrition-related care among healthcare professionals, including nurses (71, 72).

From a healthcare implementation perspective, this review identified an evidence gap between the increasing availability of food literacy assessment tools and their practical integration into routine care. Although 21 instruments were identified and several tools demonstrated potential applicability in community programs or specific disease-related populations, the pathway from assessment findings to subsequent actions remains insufficiently established. Existing studies rarely reported who should administer these tools, the appropriate settings for their use, how assessment results should be interpreted, or how identified food literacy-related needs should inform education, referral, or follow-up strategies. In nursing-related contexts, food literacy assessment should therefore be considered a supportive source of information rather than a substitute for nutritional assessment or professional dietary management.

Therefore, the current challenge is not the availability of food literacy assessment instruments, but the limited evidence regarding implementation pathways that connect assessment findings with appropriate healthcare responses. Future research should extend beyond instrument development and psychometric evaluation toward implementation-focused studies examining feasibility, interpretation, professional roles, and subsequent actions. An assessment–action framework incorporating result interpretation, interdisciplinary decision-making, targeted support, and outcome evaluation may provide a potential approach for translating food literacy assessment into context-sensitive healthcare practice, including nursing-related settings (73).

## 5 Conclusion

This scoping review mapped the development, cultural adaptation, and potential applications of adult food literacy assessment tools over the past decade. The findings indicate that available instruments have expanded beyond individual knowledge and skills to incorporate broader behavioural, social, environmental, and food-system-related dimensions, although many tools continue to primarily assess individual capabilities and provide limited information regarding how food literacy is applied within everyday contexts. Cultural adaptation strategies were descriptively categorized into language and semantic adaptation, structural and measurement adaptation, and conceptual and value embedding. While linguistic adaptation and psychometric evaluation are frequently reported, culturally embedded dietary meanings and context-specific food practices remain less explored.

From a healthcare implementation perspective, this review identified an evidence gap between the development of food literacy assessment tools and their integration into practice, including nursing-related contexts. Although some instruments have shown potential applicability in community programs or specific disease populations, evidence linking assessment findings with interpretation, education, referral, follow-up, and other care processes remains limited. Future research should therefore prioritize implementation-focused studies and develop and evaluate assessment–action frameworks that support context-sensitive and interdisciplinary healthcare practice.

### 5.1 Strengths and limitations

This scoping review followed the methodological framework proposed by Arksey and O’Malley and PRISMA-ScR recommendations, focusing on mapping and synthesizing existing evidence rather than evaluating study quality. A major strength was the integration of dimensional development, cultural adaptation strategies, and potential practice applicability of adult food literacy assessment tools from a nursing perspective. The reported cultural adaptation approaches were descriptively organized into three categories, providing a structured overview of current adaptation practices and remaining gaps.

Several limitations should be acknowledged. As a scoping review, this study did not assess the methodological quality or risk of bias of individual studies, and the findings should not be interpreted as comparisons of instrument superiority. The exclusion of non-Chinese and non-English studies and grey literature may have resulted in the omission of potentially relevant instruments from other cultural or practice contexts. In addition, heterogeneity among instruments regarding conceptual definitions, target populations, assessment domains, and validation approaches limited direct comparison across tools.

## Data Availability

The original contributions presented in the study are included in the article/supplementary material, further inquiries can be directed to the corresponding author.
